# Supplementary material for: Lecturers’ information literacy experience in remote teaching during the COVID-19 pandemic
Source: PLoS One. 2022 Mar 18;17(3):e0259954. doi: 10.1371/journal.pone.0259954 (PMC8932599; doi:10.1371/journal.pone.0259954)
Supplement: S1 File — (DOCX) [file pone.0259954.s001.docx]

**S1 Supporting Information. Interview guide**

Lecturers' information literacy experience in remote teaching during the COVID-19 pandemic

Individual Interviews

**Discussion Guide – Duration: 30-60 minutes**

1. Introduction (3-5 mins)

- Greetings
- Introduction to the overall project
- Purpose of the interview
- Confidentiality
- Consent process
- Individual opinion and experience (no right or wrong answer)
- Focus on the ways lecturers experience distance teaching during the COVID-19 pandemic
- Recorded audio.

2. Prompt Questions: (20-50 mins)

The following questions are illustrative of the formats to expect during the interview. This list is not exhaustive, as other questions based on the generated points by the participants, are likely to emerge. In addition, the focus is on creating a more natural free-flowing dialogue or conversation, but not a formal stilted setting. Every effort is provided to ask questions in a natural and modest approach, probably in no particular order. However, certain questions may be missed, while others newly added, depending on the conversation flow. General probe questions are also intended to stimulate further information from participants on the responses provided.

1. Can you tell me what your subject is?

Probe questions: a. How many subjects do you have for this semester?

b. How do you manage your subjects?

c. How many students do you have for one subject?

1. Can you tell me about how you deliver lectures?

Probe questions: a. What platform do you use for teaching?

1. How do you go about using the platform?
2. What features do use for teaching most? And why?
3. Can you tell me the way you engage with your students?

Probe questions: a. What works best?

b. What doesn’t work quite well at this stage?

1. What features do use for teaching most? And why?

4. Can you tell me about the information sources you use for your distance teaching?

Probe questions: a. What types of sources do you use?

b. How did you found the sources?

c. What do you like most about these sources?

d. What do you like least about these sources?

5. How would you define ‘distance teaching’?

Probe questions: a. Can you tell me the most challenging things you have when you are teaching?

b. What will you do to overcome the challenges?

General probe questions

- Could you explain that further?
- What do you mean by that?
- Why is that important?
- Could you please give me an example?

3. Summary (2 mins)

- Question & Answer Time
- Thank participant
