# Supplementary material for: Lecturers’ information literacy experience in remote teaching during the COVID-19 pandemic
Source: PLoS One. 2022 Mar 18;17(3):e0259954. doi: 10.1371/journal.pone.0259954 (PMC8932599; doi:10.1371/journal.pone.0259954)
Supplement: S2 File — (DOC) [file pone.0259954.s002.doc]

**Pengalaman mengajar jarak jauh sebagai bagian dari literasi informasi dosen FIB**

I: Di *channel* Ms teams sama di *youtube*. Nah, tapi seperti biasa jadi ada pesan sponsor kalau tolong dikunjungi *youtube*, *like* dan *subscribe*. Responnya bagus banyak juga yang mengunjungi dan *subscribe*. Hehehe

P: Hehehehe, itu dia pak kalau di teams kan ndak ada, kita ndak bisa tahu mahasiswa itu mendengarkan atau ndak. Tapi kalau di *youtube* ada jumlah *view* ya pak ya

I: He’em .

P: Betul, dan menyenangkan

I: O iya iya, betul

P: dan menyenangkan

I: Cuma kalau di ms teams itu kan setiap kali, karena itu materi belajar perminggu begitu, setiap kali saya beri *assignment* dan *assignment* itukan bisa dilihat dari *grade* ya, dari .. dari apa, eee…. Siapa yang sudah membuat tugas, ada yang terlambat satu menit dan sebagainya dan sebagainya kan ter.. terekam di di di ini, di *grade* *assignment* itu.

P: Iya iya iya, oh iya iya iya , betul betul betul pak, mantap mantap saya…

I: Saya kalau pertibangannya ini sekalian ngunggah di *youtube* itu kan pertama pikir saya bisa diakses siapapun yang .. yang berminat kan, jadi karena waktu saya hanya unggah di.. di ee…*facebook*, itu respon ada temen-temen mahasiswa dari unnes, kemudian ada juga dari unair dan sebagainya, yang dia bilang, ee..“boleh nggak saya *share* ee lagi” gitu ya saya silahkan, nah trus akhirnya, wah daripada kepalang tanggung

Saya unggah sekalian di *youtube* dan itu iniii.. apa namanya yang teknis yang ngerjain apa abram anak saya

Si si [name] itu, e e, jadi dia yang bikin yang mengedit, jadi saya, saya cuma merekam sendiri dengan hp setengah jam itu karena sekitar durasi 15 menit kan. Terus, ee… setengah jam itu selesailah, apa eee.. merekam sendiri. Udah itu di edit di ini sampai pada siap di unggah dan yang mengunggah itu apgram itu, jadi sangat terbantu untuk untuk publikasi itu.

P: Emmm, mantap mantap ..

I: Pikir saya bisa diakses lebih luas siapappun ya, dan yang kedua konten itu menurut saya kalau ada kekeliruan sana sini bisa dikirtik kan dan itu untuk memperbaiki konten itu, jadi istilahnya seperti apa ya, sebenarnya seperti publikasi juga tapi materinya materi pembelajaran, hanya kalau disitu ada kekeliruan kan, tolong di kritik, dan nanti apa, bisa diperbaiki kan, kemungkinan kekeliruan kan pasti ada ya, saya pikir seperti itu.

P: Kekeliruannya itu dalam bidang apanya ya pak ya, dalam hal apanya ya pak ya?

I: Nah, saya kebetulan , e….. apa, materi kuliah itu kan Filsafat Ilmu, dan dan kebetulan saya tim *teaching* jadi saya kebagian setelah UTS, jadi ini baru berjalan minggu ke 3 dan ke 4 ini, nah karena .. apa, konten itu saya kan pakai apa saya *update* referensi mutahir dan sebagainya, itu maksud saya referensi itu tidak referensi yang dulu-dulu banget, gitu lho … nah, ada kemungkinan karena saya memahami referensi karena itu referensi-referensi relatif baru dan sebagainya ya. Apalagi menyangkut bidang linguistik. Lalu ada, ya, terutama semiotika ya. Terus ada bidang-bidang, e.. kalau yang masalah filosofis dalam ilmu fisika, biologi, dan psikologi itukan eee… sebenarnya kalau ada cukup waktu perlu *crosscheck* untuk .. untuk sedikit belajar tentang eee…. Konflik itu, bener-bener dari fisika, dari biologi dan dari psikologi gitu, nah. Karena berhubung itu iya kan, enggak.. enggak cukup waktu dan sebagainya. Udah, apa yang saya pahami dari referensi yang baru referensi yang mutakhir itu dari ee…apa, Staffan Carlshamre itu orang Swedia ya, ee.. filsuf Swedia itu ya. Nah, udah saya luncurkan aja. Eee… apa, kan itu tidak lepas dari, dari kemampuan pembacaan saya yang asumsinya mesti ada lah di sana sini kekeliruan, salah tafsir dan sebagainya. Nah kalau itu ada umpan balik, menurut saya kan bagus ya, seperti itu.

P: Ya betul, sepakat pak, karena teks itukan tergantung kepada siapa yang membaca dan menginterpretasikan ya pak ya..

I: Betul, iya, dan itu sejauh referensi dia kan. Ada yang bisa memahami dengan bagus tapi kan ada juga yang cethek gitu ya, nah, saya mungkin masih di tataran yang cethek-cethek itu.

I: Kayag renang itu baru pemula gitu lho pak, renang itu malah ya istilahnya baru di 60-70 cm itu, hehehehe, jadi masih cethek itu.

P: Hehehe, tapi selama ini gaimana pak, selama ini maksudnya , eee… selama ini ada *feedback* atau *comment* tentang kontennya dari panjenengan?

I: Kok belum itu ya, saya buka ini belum.

P: Belum ya

I: Belum, jadi ya itu baru di tonton gitu ya istilahnya. Tapi, dari… apa, ee… respon itu , ee… belum ada yang masuk.

P: Iya, iya

I: Ya mungkin karena sebatas banget ya, karena berbahasa Indonesia, satu. Terus mungkin ceritanya lain ya kalau itu dalam Bahasa Inggris, akan e audiesnya itu akan lebih luas lagi ya.

P: Ya pak, cuman bapak kan membuat Bahasa Indonesia karena targetnya saat ini kan masih mahasiswanya bapak begitu kan ya?

I: Iya, iya betul, untuk kelas yang kita pegang ya, di di universitas. Itu ngomong-ngomong termasuk mata kuliah yang boleh dibilang ee…. Mewah. Maksudnya gini, mewah itu, untuk kurikulum S1itu biasanya tidak mencantumkan Filsafat Ilmu, karena itu munculnya di S2 setidak-tidaknya. Nah, di prodi Sejarah, itu muncul Filsafat Ilmu itu, sebagai satu mata kuliah. Itu saya bilang, e… apa namanya, ya nggak ini lah ya.

P: Kalau menurut bapak mata kuliah Filsafat Ilmu itu, e.. bagaimana pak kalau diterapkan di S1, pak?, tadi bapak bilang kan itu seharusnya S2, saya juga pernah dengar begitu soalnya. Kira-kira kalau untuk S1 bagaimana pak kira-kira, untuk kita baru menghadapi anak yang baru lulus SMA gitu ya, ketika diberi itu, itu apakah bisa, nggak tahu, menurut bapak bagaimana? Tepat nggak pak?

I: Kalau saya melihat sisi bagusnya itu, e.. pengenalan sih, pengenalan tentang e… apa, ya terkait dengan konsep-konsep kelimuan pada umumnya, e.. paradigma itu ya. Jadi setidak-tidaknya kan kita mengenal ada paradigma *positivisme*, paradigma hermonetik, dan paradigma fenomenologi itu. Bahwa masing-masing cenderung juga ada yang jadi e.. apa, paradigma di ilmu-ilmu alam tapi juga di ilmu sosial dan ilmu humaniora terutama hermonetika dan fenomenologi. Terus e.. juga pengenalan dari metodologi itu sebagai satu e.. metodologi penelitian. Jadi *positivisme* sebagai salah satu metodologi penelitian lalu hermonetika atau interpretasi itu juga sebagai satu metodologi penelitian dan fenomonologi itu. Jadi, e.. ini saya kira memberi satu wawasan, untuk e.. apa namanya pembelajar, untuk mahasiswa S1 setidak-tidaknya, mungkin kalau saya, e.. apa namanya .. mengunduh dari materi pembelajaran di malah di lingkungan e… pendidiakan SMA ya, misalnya *high school* kelas, materi kelas 11, jadi kelas 2 SMA kan?, itu ada e.. *logic and methodology research* di India.

I: Untuk Kementrian pendidikan India, e…kurikulum kelas 11 itu ada mata pelajaran *logic and methodology research*

I: Jadi itu pengenalan yang lebih awal dari apa .. berfikir logis dan lalu untuk mengantarkan pada tadi paradigma metodologi penelitian itu kelas 11 lho, lha di kita e… Filsafat Ilmu aja, hanya di beberapa prodi katakanan dari sekian banyak di tempat kita misal ya. Tapi, kalau S2, S3 kan memang sudah biasa itu ya, Filsafat Ilmu.

I: Tapi saya kira sisi bagusnya itu memberi wawasan ya.. tentang ilmu itu sendiri sebagai satu kegiatan utama ya, terutama disini adalah penekanan pada penelitianya, dan lalu e..

I: etos dari semangat masyarakat ilmiah itu yang Robert Merton itu ada *universalism, communism, disinterested*, dan *skepticial organizedsm*. Nah itu kan memberikan wawasan ya, memang tidak semua akan menempuh jalur akademik, tetapi dia sudah punya wawasan tentang kerja-kerja di bidang akademik itu utamanya adalah penelitian, lalu dia dikenalkan dengan paradigma itu, dengan paradigma untuk keilmuan dan lalu metodologi penelitiannya, gitu…, ya pengantar sih pak.

P: Bahkan saya berfikir bahwa meskipun tidak banyak yang akan mengambil akademik tapi kalau kita melihat lebih jauh sebenarnya itukan landasan berfikir ya pak ya?

I: Iya betul

P: Yang bisa digunakan diberbagai macam aspek gitu, saat nanti mereka bekerja.

I: Betul, saya pikir itu bagian dari kompetensi sebenarnya, ketika *critical thinking*.

I: Ya, masuk pada *critical thinking-*nya, ya masuk pada *analysis and* *critical thinking* sebagai salah satu kompetensi dari lulusan S1, itu kayagnya eeee…. Apa namanya, kontribusinya kesitu kalau mau sih.

P: Dan itu harusnya jadi luaran wajib pak, untuk mahasiswa. Berfikir kritis dan apa, berfikir *logic* tadi itu. Karena itu dibutuhkan di semua industri pak. Dimana pun

I: He’emm, untuk *problem solving* kan akhirnya

P: He’em, iya betul.

I: Iya betul

P: Saya kira begitu, nah ini selama udah hampir 2 bulan ini kan pak, kita kan mengajar mengajar ini kan jarak jauh dan online ini

I: Iya

P: Nah, saya melihat materi bapak seperti itu, itu bagaimana persiapannya pak?, maksudnya kayag mencari *resource* nya atau e… ya maksudnya kemudian jadi satu paket di *youtube* atau di ms teams itu ..

I: Emmm, ya memang sebetulnya persiapannya nggak mendadak sih, nggak ujug-ujug

I: Maksdunya, saya selama ini punya bahan itu ya..

P: Iya

I: Punya bahan yang bisa dibilang cuma numpuk gitu lho.

P: Emmm..

I: Tapi begitu ada covid-19, itu buat saya seolah-olah itu, e.. berkah gitu lho.

P: Mantap, mantap..

I: Jadi, bahan-bahan yang menumpuk itu, akhirnya kan saya berfikir e.. dengan kuliah *daring* dan sebagainya kan terjadi perlakukan terhadap kelas ya..

P: Iya, iya

I: Dan perlakukan itukan memerlukan adaptasi

P: He’em

I: Nah, menurut saya adaptasinya itu signifikan kalau kita lihat dari komponen pembelajaran. Dari komponen pembelajaran 3 itu ada silabus, ada model, dan lalu yang ke 3 sarana pembelajaran. Ok, kalau kita bicara silabusnya tetap, silabus itu kita tetap.. tetap pakai, tetap mengacu ke itu gitu lho.

P: He’em

I: Tapi, yang adaptasi signifikan kan yang model pembelajaran dan sarana pembelajarannya.

P: Iya

I: Nah, model pembelajarannya e.. kita, e.. apa namanya pakai *daring*, via ms teams misalnya itu.

P: Hemmm

I: Nah sarana ini, yang lalu e… apa, ya dari bahan-bahan yang sudah ada itu akhirnya jadi materi kuliah, ya itu dari teks ya, ada yang pdf lepas-lepas lalu ada yang *full* gitu.

I: Lalu juga PPT. Nah, kalau teks dan PPT itu selama ini memang sudah saya pakai. Itu ber…. Ya selama bertahun-tahun ini dalam kelas itu kan.

I: Nah, dalam hal ini yang menurut saya strategi adaptasi itu video, video yang terus, aduhh iseng-iseng saya unggah lah, tadinya di *facebook*

P: Ya

I: Lalu dapat respon, udah palang tanggung di *youtube* sekalian. Jadi adaptasi yang baru menurut saya melalui video itu, video ini kan saya unggah juga di ms teams lalu *youtube* ya.

I: Tapi saya baru tahu dari bapak tadi, kalau di.. di *youtube* itu malah *free* ya untuk mahasiswa ya.

P: Iya, iya ada beberapa paket kuota itu yang memang bisa akses medsos termasuk *youtube* pak, itu. Itu ndak mengurangi kuota mahasiswa

I: Mereka ya, Nah, di Ms Teams misalnya durasi video saya kan sekitar 15 menit

I: Tambilan di Ms kan juga nggak bisa bagus ya..

I: Maksudnya tergantung jaringan, jadi saya yang saya apa, yang selama 2 bulan ini kan kadang jengkel itu kendala jaringan

I: Kadangkan jaringan di mahasiswa e…, kebetulan kormat itu kan sekaligus dia juga membantu untuk mengorgan, megorganisi ya ..

P: He’emm.. he’em..

I: Jadi, begitu ms teams kita buka, e… kormat ini lalu ini, tapi kadang-kadang mahasiswa emm melaporkan, maaf pak, apa, ini kami kesulitan jaringan. Pertama itu, kedua, kadang dari pihak saya sendiri.. ee… saat-saat tertentu mbukanya susah sekali, sementara mahasiswa sudah siap.

P: Hemmmm…

I: Nah, dengan kasus seperti itu, tadi kormat itu menghendel dia, menorganize berlangsungnya *meeting chat* itu

P: Oh… mantep ya

I: Iya, *meeting chat* itu berlangsung dan sebagainya, saya nanti menyusul kalaupun kalaupun apa, gagal sampai.. sampai.. e… apa, kegiatan berjalan. Mereka tetap bisa kirim recordnya itu, recordingnya itu ke Ms Teams

P: Emmmm,..

I: Jadi memang kerjasama saya kira, ada saatnya saya jengkel karena ini udah se.. se.. apa, 10 menit, 20 menit tapi dari via mahasiswa kan terutama yang persentasi kelompok kan mereka harus, ya harus .. apa namanya, satu kelompok itu harus siap dan lain sebagainya. Gitu..

P: He’emm.. he’emm..

I: Nah, kadang saya sendiri juga jengkel karena mbukak tidak selalu, tidak selalu e… apa namanya, langsung bisa berhasil ya, nah… tapi, kesulitan dari dari saya itu bisa di ini, bisa dibantu dalam hal ini mahasiswa itu menghendel kelasnya itu untuk tetap berlangsung. Tetap lancar seperti itu.

I: Jadi memang jaringan ya, dan jaringan ini juga juga kalau saya ya, sangat-sangat tergantung paketan internet, kebetulan saya kan e… beli paketan itu 28 giga itu yang dia bilang untuk paket satu bulan tapi karena keperluan e.. kuliah *daring* yang saya praktis memegang 19 kelas.

I: Rata-rata sehari itu 4 kelas

I: Ya jadi on air gitu, rata-rata sehari 4 kelas itu paket 1 bulan Alhamdulillah 2 minggu habis.

I: Jadi harus isi ulang.

I: Kayag tadi itu *workshop*, *workshop* .. emmmm… iya belum selesai tapi paketnya habis dan saya keluar sebentar terus ini udah gabung lagi ke *workshop* itunya.

I: 2 minggu untuk 28 giga itu ya buat saya. Emmmm….. ya ..

P: 28 giga itu ya besar lho terhitungnya ya pak ya

I: Lumayan

P: Lumayan ..

I: Untuk paketan [nama provider] itu

P: Mahal lho itu

I: Kalau untuk paketan [nama provider] itu nominal sih nggak mahal banget cuma 165 ya.. dibandingkan dengan 12 giga selama belum ada kuliah daring kan saya hanya 1 bulan itu pakai 12 giga, 12 giga itukan sudah seharga 100 ya,

P: Emm… iya, iya iya

I: 100 itu ya, nah ini 28 giga 165. Ya

P: Berarti selama sebulan ya 56 giga ya pak ya? Paling tidak ya?

I: Iya pak iya, segitu.

P: Emm..

I: Saya pakai, pakai [nama provider] yang cukup sebenarnya jaringannya kan ini ya, luas gitu ya .. he’e…. Saya nggak tahu dengan pengalaman yang dengan operator lain ya.

I: Jadi, jadi kendala jaringan satu juga paketan internet. Nah, saya juga ee… mengasumsikan bahwa masalah jaringan dan paketan ini juga menjadi masalah yang dihadapi temen-temen mahasiswa

I: Ada satu kali kelas berlangsung, dia harusnya presentasi itu ya, terus dia, e.. dia Bilang di WA, kan setiap kelas saya bikin grup di WA untuk berkomunikasi di situ. Dia bilang “Maaf pak, saya ini udah keluar rumah lho ini lari-lari di tengah sawah tapi belum dapat juga jaringannya”.

I: Saya tidak tanya anda dimana dan sebagainya, saya hanya bilang “oh sudah kalau begitu, cepat balik ke rumah, nggak usah lari-lari jauh-jauh lah. Hahahaha.

I: Iya, itu sampai nyari jaringan di tengah sawah,

I: Saya bayangkan kan posisinya kira-kira agak jauh dari tower. Hehehe

I: Agak jauh dari tower ya..

P: [nama provider] saya jelek pak, he’em..

I: Ohh gitu …?

P: Iya, saya pakai [nama provider], istri juga pakai telkomsel dan jaringannya nggak ba... Apalagi kalau misalnya di pakai *tethering* di laptop. Nah itu sudah.. makin lemot

I: Oh ya, iya . *tethering* ya.. he’em ..

P: Kalau pak Selamet, pakai *handphone* atau pakai laptop pak? Selama mengajar pak?

I: Pakai laptop pak

P: Pakai laptop ya, iya iya ..

I: He’e.. iya

P: *Tethering* to berarti pak.

I: Iya, he’em. Handphone… oh iya kalau handphone enggak, saya nggak pakai. Handphone itu untuk WA grup kelas itu, terus untuk ngeshare *QR Code* itu ya, *QR Code* itu memang saya *share* lewat WA grup itu.

P: Emmm.. iya iya iya..

I: Nah nanti kalau waktunya lewat trus pada *list* kan, yang gagal SSO ya saya klik itu. Ya saya nggak ambil pusing

P: hehehehe

I: E… apa, begitu dia nggak ini, saya klik i hadir aja. Ngapain juga harus anu..he’em.

P: Karena..

I: Saya memahami tadi..

P: Betul.. betull..

I: Yang lari-lari ke sawah itu lho, pak.

P: Hehehehe, iya pak, hati-hati kejeglong.

I: Hehehehe

P: Hehehehe, iya. Karena kan kuliah online ini kan juga bukan salahnya mahasiswa gitu. Jadi kalau ada yang kesulitan absen ya bukan salah mereka, hehehehe.

I: Betul, ya sebenarnya ini kan bukan, bukan *by design* kan tetapi menurut saya *by accident* kan?

P: Iya pak, he’em..

I: Nah, kalau *by design, by design* itu ya kita belajar dari pengalaman UT, UT itu yang sudah selama ini iya kan dengan apa, *remote learning* itu kan memang mengembangkan di, di kuliah dalam jaringan ini

P: He’em he’em ..

I: Dia, dia sudah mendesain dengan sedemikian rupa, nah kita ini kan *by accident* ya.

P: Iya, iya iya..

I: Tapi saya pikir, ya ada bagusnya juga sih.. jadi kita tertantang untuk berkreasi.paling enggak

P: iya, iya iya..

I: karena saya bayangkan kalau keadaan normal-normal saja, saya pikir kita tetap e… kelas tradisional itu, begitu ya..

P: iya, iya iya..

I: Begitu pak

P: Betul. Berarti menurut bapak… kuliah *online* ini juga ada baiknya ya pak ya, meskipun dengan agag sedikit terpaksa, karena kan memang terpaksa karena keadaan ya.

I: Ya Saya, me.. apa namanya, persepsi saya positif ya, karena emm …. Kita dikondisikan untuk menggunakan dalam hal ini adalah menggunakan emmm IT ya, dan, dan kita juga harus belajar eee…. Untuk itu, e… apa namanya, mulai dari model pembelajarannya, jadi dan tadi dan juga mengembakan materi kuliah dan sebagainya.

I: Untuk em.. *time a lotment*, 2 SKS 100 menit kan nggak pernah sampai habis itu, kecuali memang saya pegang di mata kuliah di prodi itu kan hanya 7 kelas sedangkan 2 kelas itu di luar fakultas.

I: 12 kelas itu terutama adalah teknik dan ada satu kelas di di agribisnis, tapi 11 kelas yang lain itu ada teknik elektro, teknik mesin, teknik sipil dan teknik kimia. Nah mereka dari awal ee… memang saya rancang itu hampir semua itu presentasi.

I: Jadi ketika harus, e.. apa, belajar dari rumah itu masuk-masuk minggu awal presentasi. Nah, tapi, ee… uniknya menurut saya temen-temen mahasiswa ini bersemangat gitu lho, saya setiap kali ingatkan kita sepakati ya, e… apa? Ee, presentasi untuk setiap kelompok itu 30 menit dari waktu 100 menit

I: Tapi nggak ada yang cuma 30 menit, jadi tetap berlangsung dengan diskusi sampai waktunya habis. Itu pengalaman saya dengan, dengan kelas-kelas di teknik sipil

I: Sedangkan untuk kuliah itu sedapat mungkin 15 menit, nah itu ngggak berlaku untuk kelas-kelas kami yang 11 kelas tadi di 4 kelas di teknik elektro, 4 kelas di tekim, 1 kelas di teknik mesin dan 1 kelas di teknik sipil. Nggak ada kuliah 15 menit, mereka habiskan itu waktu kalau 100 menit ya bener-bener presentasi 100 menit. Jadi mereka kuat di infrastrukturnya kayagnya, ya..

I: maksud saya kuat di, di apa namanya, eee… di paketan internet itu lho.

I: Cuma tadi, ada satu cerita maaf, cerita yang harus lari-lari ke sawah itu untuk mahasiswa agribisnis, kalau teknik nggak ada keluhan nggak ada kendala

I: Itu, mereka jalan dengan lancarnya pak.

P: Jadi permasalahan untuk sinyal dan jaringan itu ndak terjadi di teknik ya pak ya?

P: Kayagnya mungkin karena WA ini sudah 30 menit mungkin. Hehehe. Jadi…

P: Mungkin

I: Memang ada kayag timernya itu lho .. tut.. tut.. gitu

P: Anu, pak Mohon maaf, tadi berarti untuk untuk masalah sinyal jaringan tadi tidak terjadi untuk mahasiswa [nama fakultas] ya pak ya?

I: Mereka enggak, mereka lancar-lancar saja

I: Itu beda dengan kelas-kelas yang saya pegang di prodi [nama program studi], iya, jadi ee.. tidak masalah dengan dengan kendala jaringan, dengan itu..

P: Maaf kembali ke penyediaan materi bapak praktis nggak ada masalah karena emang mengumpulkan bahan-bahan sebelumnya yang sudah ada pak ya, intinya diolah lagi ya?

I: Iya betul pak. teks dan PPT sudah ada, hanya yang baru itu bikin video itu, he’em.

P: Formatnya pak ya, yang baru berarti ya?

I: Iya betul, itu betul formatnya baru tapi bahannya sudah ada sih.

P: Iya, iya iya.. Pak, jadi ee…. Ada kendala ndak pak selain sinyal saat berinteraksi dengan mahasiswa saat menggunakan teams selama ini ?

I: eee…. Emmmmm… ininya apa ya pak, apa namanya emm… persisnya bagaimana?

P: Selain kendala sinyal, apakah selama berkuliahan dalam mahasiswa ada kendala lain pak? misalnya, ada yang e…, ada yang misalnya yang aktif hanya satu kelompok saja atau misalnya ada kendala lain?

I: Kalau, eee.. apa? Aktivitas dari dari mahasiswa itu, ini terkait dengan kelas-kelas yang saya pegang di fakultas [nama fakultas] dan [nama fakultas] itu memang ee… dengan presentasi kelompok dari awal kita rancang bahwa yang aktif itu tidak hanya kelompok yang presentasi, katakana kelompok yang presentasi itu kelompok 2, tetapi melibatkan moderator itu dari kelompok 1

I: Iya, N kurang satu gitu, lalu notulis itu dari N 1 dari kelompok 3

I: Lalu, yang mendiskusikan diskusian itu selain kelompok presentasi, selain kelompok yang ada yang bertugas moderator dan yang notulis itu harus menjadi diskusen ya..

I: Jadi memang kami rancang dari awal itu membuat kelas itu aktif, tidak hanya, jadi waktu presentasi tidak hanya kelompok presentasi yang punya gawe, enggakk.. tapi semua menjadi terlibat, semua menjadi berkepentingan gitu lho. Dan responnya…

P: Rancangan ini sudah dibuat sebelum ada pandemi ini atau pas mulai kuliah online ini pak?

I: emm… jauh sebelum itu sudah saya terapkan bertahun-tahun pak.

I: he’em, nah, begitu ada pandemi, ya itu rillnya saya hanya duduk manis mengikuti ini dan nanti diakhir presentasi memberi komentar dan sebagainya

I: Tetapi praktis, kelas itu jalan, bahkan ketika saya terkendala dengan jaringan dengan di handle kormat kelas itu jalan.

I: karena dulu dirancang membuat kelas itu aktif, iya kan? Dan bisa mengatasi ketika untuk selama daring ketika dari dari dosen kesulitan untuk akses jaringan, untuk akses ke Ms Teams lamaa… begitu

I: Mereka minta ijin, bapak kami mulai presentasi ini, silahkan nanti saya nyusul, saya bilang gitu.

P: Inisiatif dari mereka sendiri ya pak berarti ya?

I: Dari mereka, saya juga salut karena kesadaran mereka sangat tinggi.

I: Termasuk untuk mengingatkan ya .. sehari sebelumnya, bapak kita besok ada kelas untuk melanjutkan presentasi

I: Jadi setiap kali, kormat itu bertanggungjawab untuk mengingatkan, lalu dia menghendel kalau tadi ada ada kendala yang dihadapi dosen maupun temen-temen gitu lho.

I: Jadi, itu pengalaman, pengalaman saya. Kalau saya boleh ceritakan pak.

P: iya terimakasih pak

P: Menurut bapak, apa yang merasa terbatasi pak? Dengan kondisi seperti ini?

I: Betul, yang terbatasi adalah *seeing contact* pak, kontak mata

I: ee… karena kita tidak bisa datang hadir berhadapan berhadapan secara ee… apa, secara langsung dengan mahasiswa.

I: dan itu di WA grup misalnya. Saya sudah jalan minggu, minggu lalu sudah mulai eror pak.. pak..

P: hehem

I: Jadi saya cerita gini, erornya. Saya menghendel memegang 19 kelas

P: Luar biasa..

I: satu kali saya mau bikin tugas itu adalah, ee… tugas.. tugas untuk *essay*, tugas itu buat kelas agribisnis tapi di Ms Teams,itu ee.. nggak tahu mungkin lagi gimana, kurang fokus dan sebagainya masuknya ke kelas elektro kelas c. Nah, terus kormatnya konfirmasi, pak, ini nanti kami ngirimkannya hanya ke Ms Teams atau juga di di email juga untuk tugas ini. Saya ngomong, lho.. rasanya saya nggak ada tugas untuk kelas ini. Tolong untuk menyegarkan ingatakan saya, tugas apa yang saya berikan?, itu tugas bla bla bla bla gitu. Maaf itu salah kirim, tugas itu bukan untuk anda, tugas itu untuk kelas lain saya bilang.

P: Emm….

I: Lalu saya bilang di WA itu, maaf saya sudah mulai eror, capek dirumah terus, sebenarnya pengen bertemu temen-temen semua. Mereka semua bilang apa? “bapak, kami sangat kangen, rasanya nggak ada candaan-candaan lucu, nggak ada semacam itu gitu.

I: kami juga jenuh gitu, tapi kan itu manusiawi

I: karena kita tidak... tidak tampil langsung

I: Iya, menurut saya, eee… apa pak, ee.. intensitas ketersampaian pesan itu untuk daring tidak sebesar ee… kuliah langsung, kuliah tatap muka.

P: Sepakat

I: Iya, kalau kuliah tatap muka, pesan kita bisa.. bisa lebih jelas ya, karena pesan verbal ya, dan dan langsung tadi, tatap muka. Tetapi dengan daring ini, ya lalu saya juga ini aja, sudahlah kalau memang tidak bisa tafsirnya penuh, ya paling nggak 80-70 %, kalau pesan itu sudah tersampaikan saya pikir itu sudah bagus.

I: Iya, jadi memang tidak menargetkan ee.. maksimal.

I: Iya, karena tadi, beda ya kalau ketemu langsung itu kerasa ya, itu misalnya itu banyak ketawa-ketiwinya pak, jadi itu nggak bisa dilakukan dengan daring

I: karena itu spontan, karena mengomentari sesuatu yang ada di kelas itu dan sebagainya

P: Iya

I: Nah kalau dari *daring* kita nggak tahu bagaimana, atau sedang apa mahasiswa itu, jadi nggak bisa merespon dan sebagainya.

I: keterbatasan itu adalah keterbatasan dari dalam hal ini adalah kontak fisik ya dan saya rasa karena keterbatasan kontak fisik akibatnya adalah ee.. intenistas pesan yang e.. yang sampai, menurut saya intensitas pesan itu kurang dibadningkan dengan kuliah tatap muka, itu menurut pendapat saya pak.

P: Iya pak, ee…. Kalau misalnya emang mayoritas itu persentasi, berarti selama ini bapak juga menggunakan medianya Eee., *voice* atau *video call* ya pak ya, belum pernah menggunakan media lain misalnya yang apa teks *chat* itu pak ya?

I: Saya, hanya pakai *meeting chat* pak.

I: *Meeting chat*, iya, jadi tidak *streaming*, karena pertimbangannya itu tadi pertimbangan beban untuk … kuota itu ya..

I: Saya hanya pakai *meeting chat*, menurut saya *meeting chat* itu lebih hemat ya,..

I: Karena tidak perlu menampilkan ee… anu kan, apa ?

P: Video

I: Jadi, saya hanya menggunakan *meeting chat*, itu saja sudah.. sudah satu langkah dari senior saya yang pegang dari awal semester sampai tengah semester itu dia tidak lewat *meeting chat* tapi lewat, hanya lewat apa, ee.. *channel*. Jadi mengunggah materi, lalu mendiskusikan, eee… lalu, itu hanya, hanya lewat *channel* aja.

P: Emmm…. *Channel*, Oke. *Channel* itu berarti hanya menggunakan teks gitu pak ya? Ngetik gitu pak ya?

I: Hanya menggunakan teks, jadi kalaupun menjawab “silahkan berdiskusi”, gitu ya, terus nanti diskusi itu apa, hasil diskusi dikirimkan, nah dikirimkan itu dikirmkannya muncul di *channel* jadi bukan *assignment*.

P: Oh.. iya iya iya..

I: Kalau assignment kan sampai pada masing-masing individu ya, atau semua yang termasuk kelas itu ee… kita tahu..

P: Emmm..

I: Kita, kita, bisa bisa melihat historynya, nah tapi senior saya yang awal semester itu dia selalu hanya menggunakan *channel*.

P: Emm…

I: Di *channel* itu hanya teks ya.. dan teks itu ya nggak ada suara ya..

P: Iya

I: Hanya tulisan itu

P: Betul.

I: Silahkan di diskusikan nanti setelah di diskusikan dikumpulan apa, hasil yang di disiskusikan ya udah muncul di apa namanya *channel* itu “ini hasil diskusi kelompok kami, point 1, 2, 3 dan seterusnya,” sudah itu saja.

P: Emm..

I: Lalu, ee…. Setelah gentian saya yang pegang, saya minta kormat tolong bikin WA grup, karena di WA grup ini pesan-pesan akan disampaikan dan *QR Code* akan saya *share* di WA grup itu.

P: Emmm..

I: Jadi tidak ke Ms teams, untuk memishakan presensi dengan ee.. apa, perkuliahan.

P: Emmm….

I: Tapi ya itu, kalau kelewat waktu, saya akan bantu untuk klik untuk kehadirannya, itu selalu ya..

I: Kalau saya tidak tidak akan keberatan untuk itu

I: ya sekali-sekali, emmm.. video itu dibuka untuk saling menyapa, apa kabar semua?, selamat pagi. Saya perlihatakan tampang saya akhirnya pak.

P: Hehehehe…

I: ya sesudah itu kan video di tutup, kita presentasi dan sebagainya itu dengan *meeting chat*.

P: Iya, iya iya pak. Kemudian bapak, ee… yang terakhir ini emmm… menurut bapak setelah kuliah selama ini, pengalaman bapak ini, kita-kira yang berhasil apa ya pak ya?, misalnya entah itu yang di kuliah kelas ndak ada tapi di *online* ini bisa tercapai, atau mungkin di kuliah juga ada tapi di *online* juga ada. Jadi misalnya kira-kira maksud saya begini, apakah ada faktor, ada aspek positifnya gitu lho pak, di kuliah online ini, kira-kira apa yang berhasil selama ini gitu pak?

I: Eemm…Menurut pengalaman saya pribadi, satu hal yang bisa saya katakan. Apa yang bisa dikatakan berhasil dalam kuliah *daring* itu, sejauh ini menurut saya adalah bahwa materi kuliah dalam bentuk teks, dalam bentuk PPT dan dalam bentuk video itu lebih bisa tersampaikan dengan optimal.

P: Iya

I: Dibandingkan dengan kuliah tatap muka, kuliah tatap muka, untuk teks dan PPT itu ee… ya sudah siap, tapi dalam hal ini memang, memang tidak ee.. belum, belum membuat video ya

P: He’em ..

I: Nah, maksud saya, buat saya itu buat saya bahwa materi kuliah itu disampaikan bisa penuh, kita tidak hanya tidak hanya *delivering letters*

P: Iya, iya

I: Iya, pakai oral, pakai oral itu tetapi subjek material itu kita sediakan secara penuh.

P: Iya, iya iya

I: Nah, di sisi lain, kalau saya boleh berasumsi untuk eee…. Temen-temen dosen, yang bisa jadi selama perkuliahan langsung tatap muka, eee… kemarin-kemarin itu, katakana tidak menyediakan materi kuliah secara penuh, eee… berkahnya dari *daring* ini adalah mau tidak mau semua dosen dituntut untuk menyediakan materi kuliah penuh, dan itu untuk disampaikan pada mahasiswa. Jadi e… saya melihatnya itu, bahwa materi kuliah mau tidak mau harus disediakan secara penuh

P: Emmm.. iya

I: yang kemarin selama kuliah tatap muka, itu mungkin ada porsi tadi ee.. *lecture* yang mungkin lebih besar porsinya daripada tadi misalnya penyediaan materi bilah teks, PPT, video dan sebagainya.

P: Iya , iya iya

I: Tapi dengan eee…. Kuliah *daring* ini mau tidak mau kita dituntut untuk menyediakan materi kuliah itu ee… penuh, untuk disampaikan kepada mahasiswa

P: Iya, iya iya..

I: Itu sih pak, mungkin ada yang lain? Mungkin bapak bisa memancing eee… apa lagi begitu ya…hehehe

P: iya pak. Itu materi yang dimaksud bapak secara penuh itu apakah memang di taruh dimanapun, di Ms Teams, di Youtube sampai pertemuan ke 15 atau bagaimana maksudnya?

I: Betul.

P: Ohh… Oke, iya iya..

I: Untuk teks ya, baik *word* maupun pdf, PPT dan video itu saya unggah di ms teams di *channel* itu bahkan sampai pada materi perkuliahan yang akhir

P: Emmm…

I: Iya, jadi materi perkuliahan setelah UTS itukan 8, 9, 10, 11, 12, 14, iya minggu ke 14 itu saya sudah unggah

P: Oh… iya iya iya..

I: Jadi setiap eee… apa, kali perkuliahan tinggal masuk ke *channel* itu aja

P: Oh.. iya iya iya…

I: eee.. katakana kemarin, ee.. minggu ini, kami bertemu di *channel* 10 karena ini materi minggu ke 10, tapi materinya sudah ada bahkan sampai materi minggu ke 14 itu sudah .. sudah tersedia di ms teams itu, di *channel* itu, begitu pak

P: Mantap, Itu memberi kesempatan mahasiswa untuk belajar, untuk mempersiapkan diri ya pak, sebelum ketemu di ms teams

I: Betul, betul… mereka bisa baca, baca dulu, ya..

P: He’em..

I: Karena setiap kali, eee… apa, di akhir pertemuan saya bilang minggu depan kita mendiskusikan eee.. materi ini ya,

P: Iya, iya iya

I: Jadi maksud saya ada waktu untu membaca dan kalau ketemu kita mendiskusikan. Kalau materinya udah ikuti aja itu *youtube* cuma 15 menit

P: Mantap, mantap

I: Tapi adakan, banyak yang masih perlu penjelasan dan lain sebagainya. Nah itu, kita ketemu di perkulihan itu untuk mendiskusikan itu, begitu lho..

P; Iya, iya iya.. keren, keren, keren

I: Begitu pak.

P: Keren pak.

I: Saya kira itu ya, strategi adaptasi ya..

P: Betul, iya iya iya iya..

I: Hehehehe

P: Ee.. mungkin ini yang beneran terakhir pak, hehehe… kira-kira..

I: Iya, masih akan hehe.

P: Misalnya pandemi ini terus berakhir pak misalkan, mudah-mudahan berakhir, ini selesai, apakah kedepannya bapak merasa terinspirasi dengan model seperti ini pak, untuk di implementasikan di metode mengajar pak kedepannya?

I: Ee.. maaf gimana pak, diulang lagi!

P: ya pak, nanti misalnya setelah ee… pandemi ini berakhir

I: Setelah pandemi ini berakhir, ya oke..

P: Ya, apakah bapak merasa terinspisasi untuk tetap melakukan kuliah *online* ini atau bener-bener kembali ke kelas seperti semula, pak? Kira-kira, kira-kira..

I: Nah.. itu tergantung kebutuhan ya pak say akira, prinsipnya tergantung kebutuhan, kebijakan dari institusi kita kan ya..

P: Iya..

I: Jadi, katakanlah nanti eee… Mei ini berakhir gitu, kalau saya sih mengharapkan tetap ada kesempatan tatap muka

P: Iya,..

I: Karena tatap muka itu tidak sepenuhnya tergantikan

P: Iya

I: Tetapi, kalu kondisinya tidak memungkinkan dan dan perkulihan itu katakan sampai tahun ajaran baru, ya..

P: Iya

I: Mahasiswa baru tahun 2020 nanti semester gasal misalnya, pandemi masih belum berakhir, itukan kemungkinannya perkulihan juga akan berlangsung penuh secara *daring*

P: Iya , iya iya

I: Iya, buat saya, nanti tinggal, tinggal apa, mengembangkan lagi, improviasai lagi, berkereasi lagi gitu..

P: iya, iya iya..

I: Nah, jadi tergantung kebijakan dari dari Institusi dan tergantung kebutuhan itu.

P: Iya, iya

I: Tapi secara pribadi, menurut saya ee… peretemuan tatap muka itu tidak sepenuhnya bisa tergantikan ya..

P: Iya

I: Jadi

P: Iya, He’em ..
