# Supplementary material for: Lecturers’ information literacy experience in remote teaching during the COVID-19 pandemic
Source: PLoS One. 2022 Mar 18;17(3):e0259954. doi: 10.1371/journal.pone.0259954 (PMC8932599; doi:10.1371/journal.pone.0259954)
Supplement: S3 File — (DOCX) [file pone.0259954.s003.docx]

**TRANKRIP WAWANCARA INFORMAN 2**

Informan : “.. ini jadi perkuliahan tidak harus ee.. dilaksanakan secara bertatap muka tapi bisa juga menggunakan kuliah online seperti itu, tentu saja ada sisi positif dan sisi negatifnya, itu pengertian kuliah online menurut saya, itu baru pengertiannya yah belum ke kendala dan segala macamnya seperti itu.”

Peneliti : “Bapak mengajar berapa mata kuliah pak, kalau boleh tau dalam satu minggu?”

Informan : “Dalam satu minggu sesuai dengan mata kuliah yang saya ampu sebanyak sekitar 16 sampai 18 SKS kalau tidak salah.”

Peneliti : “Oke iya, itu kalau jumlah mata kuliah berarti sekitar berapaa ya pak?”

Informan : “Sekitar delapan, sekitar tujuh sampai delapan ya, mungkin saya delapan mata kuliah yang saya ajar.”

Peneliti : “Bapak menggunakan aplikasi apa pak saat mengajar?”

Informan : “MS Teams, MS Teams seperti yang disarankan oleh pihak fakultas”

Peneliti : “Oke, itu aja pak ya, ndak pake aplikasi yang lain ya buat tambahan?

Informan : “Iya, itu saja. Dulu si pernah sempat ingin menggunakan Kulon sambil belajar tapi ternyata aplikasi kulon itu ee.. menurut saya, saya lebih seneng MS teams karena untuk fiturnya lebih lengkap dan bisa komunikasi langsung dengan mahasiswa. Kalau kulon kan tidak seperti itu keliatannya ya, lebih ribet keliatannya kalau [nama universitas] tuh.”

Peneliti : “Bapak saat berinteraksi dengan mahasiswa metodenya bagaimana pak?”

Informan : “Tergantung mata kuliahnya, kalau untuk misalnya saya mengajar mata kuliah kanji yah, kalau kanji saya itu biasanya menggunakan PPT jadi saya tulis kanji-kanji yang dipelajari di perkuliahan itu dalam PPT terus saya.. apa namanya.. upload, share jadi melalui PPT itu saya mengajar kanji seperti itu, cara bacanya, artinya, dan sebagainya.

Tapi ada kelemahannya, kalau kanji itu kan ada cara menulisnya, nah karena di PPT terbatas jadi untuk cara menulisnya saya suruh mereka belajar sendiri lihat di buku. Tapi kalau di kelas, biasanya kalau mengajar kanji saya ajarkan cara membacanya saya juga ajarkan cara menulisnya, seperti itu.

Terus untuk yang mata kuliah listening, nah itu juga mata kuliah listening ya kan ada.. ada audio yang harus di dengarkan oleh mahasiswa seperti itu. Nah ini juga kendala, karena ngga ada fitur ya untuk bisa mendengarkan secara langsung suara audio ke mahasiswa, ee.. ini juga ada kendala kalau untuk yang materinya kita menggunakan PPT juga tapi untuk audionya saya kirim dulu ke mahasiwa untuk dipelajari dulu oleh mereka, seperti itu. Kita mengerjakan, nanti kita cek bersama-sama, seperti itu.

Kalau untuk mata kuliah mengarang sih simple, karena kan minggu ini mereka disuruh untuk membuat karangan Bahasa Jepang ee.. itu simple. Nah minggu depannya untuk pembahasannya saya juga biasanya menggunakan PPT, jadi satu persatu karangan mahasiswa itu saya tampilkan di PPT mana yang kesalahannya ee.. saya koreksi ini harus diperbaiki, seperti itu. Terus untuk mata kuliah terjemahan juga sama seperti itu, minggu ini mereka menerjemahkan suatu karangan ya.. misalnya suatu karya, minggu depannya saya bahas bersama-sama.

Terus satu lagi mata kuliah speaking, nah itu yang agak berat mata kuliah speaking karena harus ada interaksi lansung ya ee.. antara mahasiswa dengan dosen untuk bisa saling berkomunikasi, bercakap-cakap seperti itu, itu juga ada kendala karena masalah kuota. Nah kalau memakai video kan kelihatannya mereka keberatan yah, jadi kita hanya mendengarkan suara-suara saja seperti itu. Jadi kebanyakan untuk medianya kita menggunakan PPT seperti itu lebih praktis.”

Peneliti : “Banyak cara ya pak, berarti cara tergantung pada konteks materinya ya pak ya”

Informan : “He eh, iya, banyak cara sih sebenarnya iya seperti itu”

Peneliti : “Saya pengin tau, bagaimana caranya Bapak bisa menemukan berbagai macam strategi ini pak?”

Informan : “Ooh... ini karena memang disesuaikan dengan kebutuhan saya selaku pengajar pada saat mengajar satu mata kuliah tersebut, karena setiap mata kuliah itu dalam Bahasa Jepang itu mempunyai karakteristik masing-masing ya, seperti itu. Kalau misalnya untuk mata kuliah ee.. speaking, untuk mata kuliah speaking itu saya biasanya mengambil beberapa metode. Metode yang pertama saya, harus mengingat kembali kebutuhan mahasiwa bahwa ini kalau misalnya kita video terus nanti ee..nanti mereka jebol, apa namanya kuotanya, seperti itu kasian kan. Jadi kita nanti biasanya untuk speaking dengan saya berduanya itu setelah mereka mengerjakan tugas itu hanya lewat audio saja, tapi nanti mereka juga setelah selesai dengan.. apa.. setelah selesai audio dengan saya mereka harus ngirim script apa namanya percakapan yang emm.. yang mereka buat sesuai dengan tugas yang saya berikan. Nanti dikirim juga ke saya apakah tadi speaking saya tadi dengan script yang dia buat itu benar atau tidak seperti itu. Terutama untuk kelas-kelas bawah ya, kelas kelas bawah kadang ngaco kalau speaking itu grammarnya segala macamnya ngaco seperti itu. Kalau kelas-kelas atas scriptnya itu engga saya minta si mereka sudah pinter sekali langsung ngomong seperti itu. Terus untuk kanji juga variasinya banyak sih tidak hanya menggunakan PPT juga yang lain juga tugas-tugas dan segala macamnya, seperti itu.”

Peneliti : “Itu metode yang bapak lakukan sebelum pandemi atau bapak menemukan sendiri saat mengalami ini pak, kuliah online pak?”

Informan : “Ohh saya menemukan sendiri, karena saya ingin kuliah ini meskipun online harus sifatnya harus bersifat efektif yah, dalam artian satu lagi karena sekarang online kegiatan mahasiswa di sana itu saya suruh bayak mereka emm.. cari cari sendiri di internet untuk penambahan tugas tugas misalnya kanji cara menuliskan kan sekarang sudah diajarkan sama saya jadi mereka harus apa namanya harus kreatif sendiri mencari-cari di internet, misalnya kanji ini berapa coretannya terus simbolnya apa seperti itu biasnaya saya seperti itu. Kalau dulu pas sebelum pandemi ini, ya kalau missal saya ngajar kanji ya sendiri yang mengajarkan kepada mereka tetekmbengeknya ya sampai penulisan sekecil-kecilnya. Ini kuliah online ini ada kelebihannya ada sih membuat mahasiswa mereka menjadi kreatif . Jadi yang namanya mahasiswa itu kan sebenarnya dosen tidak perlu satu persatu disuapin yah, jadi mereka harus kreatif sendiri seperti itu, jadi ada gunanya juga sih mereka kreatif sendiri untuk mencari tau apa yang mereka tidak dapat pada saat di kuliah online, seperti itu. “

Peneliti : “Mantap.. saat mahasiswa diminta untuk mencari sendiri bahan di internet, sejauh ini keberhasilannya bagaimana pak ya mereka menemukan bacaan itu ya pak ya? bapak mengetahui ini nggak ya pak?”

Informan : “Iya, he eh tau banget karena misalnya untuk.. iya tau banget dan kebetulan di internet banyak sekali ternyata gampang yah nyarinya untuk masalah untuk terutama untuk yang berhubungan ke-Jepang-an, karena pemerintah Jepang itu menyiapkan web web yang bisa digunakan oleh mahasiwa untuk belajar sendiri Bahasa Jepang. Jadi lebih menggunakan metode metode pengajarannya dibandingkan dengan yang diajarkan oleh saya sebenarnya seperti itu, dan sangat efektif sekali seperti itu makanya begitu ngajar itu mereka udah tau sendiri ‘oh iya sensei ini carabelajar nulisnya seperti ini, ternyata dari web yang dari Japan Fondation dari web dari JICA ternyata ada katanya, pokoknya kanji-kanji yang saya ajarkan bagini begini begini seperti itu. Jadi lebih bagus online itu membuat mereka kreatif mencari sendiri web web untuk sebenanrya mereka bisa belajar sendiri seperti itu dari Japan Fondation, dari JICA, dari Marugoto nah seperti itu. Menarik ini sebenarnya, menarik..”

Peneliti : “Berarti secara enggak langsung artinya menemukan sesuatu yang enggak kita duga sebelumnya ya pak ya ternyata malah mahasiswa ada jalan menuju ke sana”

Informan : “Iyaa sesuatu yang tak terduga ternyata mereka jadi jauh lebih kreatif, soalnya mereka merasa kurang puas.. tidak puas dengan pengajaran online karena ada beberapa yang terbatas, seperti tadi saya tidak bisa mengajar cara menulisnya tapi mereka malah lebih kreatif sendiri mencari di websait dan ada emang di sana, penemuan yang tak terduga ini..”

Peneliti : “Ternyata penemuan yang tak teduga seperti ini meminta mahasiswa untuk kreatif dan mandiri, apakah kira-kira metode belajar seperti ini nanti akan memberikan dampak ke bapak saat nanti setelah kembali ke kampus pak, setelah kembali pelajaran normal pak?”

Informan : “Iyaa.. saya juga akan mencoba tetap mempertahankan online ee.. metode pembelajarannya yaa, jadi sekarang saya paham bahwa ternyata mahasiswa ini tidak harus selalu disuapin satu persatu, tapi nanti pas saya ngajar saya akan membuat mereka lebih kreatif lagi. Misalnya, saya tidak harus mengajarkan semuanya mulai dari cara nulisnya segala macamnya, tapi cukup mereka disuruh untuk kreatif sendiri mencari di web dan nanti mereka bisa presentasi menyampaikan seperti itu, malah itu sangat lebih menarik sekali. Jadi istilahnya Tut Wuri Handayani ya atau SCL (Student Center Learning) jadi tidak harus dosen yang satu persatu ngajarin sampai detail seperti itu. Jadi ini bagus sekali menurut saya, nanti saya juga mencoba untuk pertahankan. Dan mereka biasanya senang sih kalau disuruh-suruh untuk mencari sesuatu yang baru, misalnya kanji ini bagaimana cara penulisannya silakan cari Anda di web yang lebih menarik, apakah dari alam atau dari.. jadi mereka sebenarnya suka disuruh-suruh seperti itu.”

Peneliti : “Nah saat bapak, saya ikut senang pak mendengar mahasiswa suka dengan caranya bapak cari-cari kayak gitu. Saya jadi pengin tau ini, saat bapak meminta mereka untuk mencari sendiri itu apakah Pak Budi juga memberikan sedikit clue atau ad acara lain pak? Sehingga mereka bisa menemukan begitu akhirnya apa yang mereka butuhkan..”

Informan : Ee.. karena kebetulan ee.. untuk mata kuliah Jepang itu banyak sekali web-web yang emang dianjurkan untuk dipelajari untuk para mahasiswa dan kebetulan saya juga kan beberapa waktu yang lalu pernah training ke Jepang juga yah sekitar dua bulanan, dan saya dikasih banyak web-web yang nanti web-web itu sebagai pelengkap mahasiswa untuk mempelajari Bahasa Jepang. Nah biasanya web-web tersebut saya..apa namanya.. saya kasihkan ke mahasiswa ‘coba Anda cari di web ini’ misalnya untuk belajar bagaiamana cara belajar kanji bagaimana cara belajar speaking untuk pengayaan, untuk pembelajaran mereka seperti itu. Tapi ada yang lebih kreatif lagi mahasiswa malah lebih tau dari pada dosennya, mereka udah tau sekarang web-web mana yang perlu mereka cari, seperti itu tuh yang menariknya ya maklum ya anak milenia ya jadi tau. Satu lagi misalnya kanji kadang-kadang mereka lebih tau ‘sensei kata ini menurut menurut ini kayak gini lho sensei begini begini begini’, jadi kadang-kadang mereka lebih tau dari kita.”

Peneliti : “Mereka itu punya kelebihan eksplor pak”

Informan : “Nah eksplornya luar biasa..”

Penelitia : “Terus satu lagi bapak, untuk mempersiapkan materi pak sekarang, dalam masa-masa Work From Home gini cara bapak mengumpulkan atau mencari materi untuk disampaikan ke kelas bagaimana pak?”

Informan : “ Oh kalau untuk materi, saya mengacu pada Bahasa Jepangnya itu kyoang yaa, kyoang itu mungkin semacam silabus yah jadi kita sudah punya silabus untuk 15x pertemuan dan dalam setiap silabus sudah jelas misalnya kita menggunakan media pengajarannya apa, worksheetnya apa, temanya apa segala macamnya apa seperti itu. Misalnya seperti kanji, misalnya kan ada kita punya bukunya juga buku kanji misalnya bab 2, bab 3, bab 4, bab 5 tiap bab kan ada seperti itu. Jadi kalau untuk misal mata kuliah kanji ee.. agak repot juga sebenarnya untuk.. untuk online tersebut kan harus selalu bikin PPT kan ya, jadi misalnya hari ini saya ngajar bab 7 jadi semua kanji yang ada di bab 7 itu mau tidak mau saya harus tulis, ketik di PPT. Misal ada 20 kanji di PPT ya nanti kita ajarkan lewat PPT, tapi pas sebelum online itu saya tidak perlu bikin PPT karena saya bisa langsung ngajar kanji tersebut pakai whiteboard, cara nulisnya, cara apa.. cara bacanya, artinya segala macamnya, seperti itu. Paling yang lebih menarik kadang-kadang saya juga eksplor nyari-nyari di internet beberapa materi yang biar nanti mahasiswa biar lebih seneng untuk belajarnya. Misal pakai gambar-gambar, misalnya kanji.. kanji langit yah saya nanti gambar kanji bintang di sana saya nyari-nyari gambar bintang, ini kanjinya seperti apa baru dicari seperti itu, biar leih menarik seperti itu. Padahal pada kuliah biasa saya tidak melakukan metode seperti itu hehehe, jadi lumayan tambahan kerjaan hahaha.”

Peneliti : “dan akhirnya juga menambah sumber informasi buat bapak sendiri ternyata ya..”

Informan : “Iya betul.. jadi saya juga mau tidak mau banyak sumber informasi baru yang saya dapat seperti itu.”

Peneliti : “itu sering menemukan nggak pak saat mencari Cara-cara menulis kanji mislanya atau yang lain, sering menemukan di internet?”

Infoman : “Alhamdulillah, sering.. sering nemu karena saya mempunyai beberapa website yang tadi saya sebutkan dari Japan Fondation, dari Marugoto, dari banyak sekali Jepang itu banyak sekali membuka web-web untuk dipelajari oleh pembelajar Bahasa Jepang seluruh dunia, dan itu sangat menarik sekali, sangat apa.. efektif seperti itu. Tapi semuanya pakai Bahasa Jepang jadi tidak ada yang pakai huruf latin, jadi yang bisa hanya pembelajar Bahasa Jepang saja.”

Peneliti : “Berarti untuk penyiapan materi relative bapak tidak ada masalah ya..”

Informan : “Iyaa..”

Peneliti : “sekarng kita bicara yang paling menyenangkan yaitu kendala, menurut bapak yang menyulitkan yang mmebuat Pak Budi kurang puas itu apa pak? Untuk kuliah online ini pak..”

Informan : “Kendala ya.. ee.. kalau kendala pertama ini ya kendala pertama yang paling deep problem itu menurut saya itu yang namanya kuliah online itu kita butuh sinyal butuh kuota dan segala macamnya seperti itu. Kendala untuk mata kuliah yang spesifik seperti yang misal butuh audio butuh visual ya kebanyakan masalah sinyal, banyak mahasiswa yang mengeluh karena daerah tempat tinggalnya itu sinyalnya engga ada jadi kita ngga bisa komunikasi. Komunikasinya cuma lewat ketikan itu loh, lewat tulisan itu loh.. lewat chatting yah, masa harus conversation pake chatting kan nggak seru yah, kalau conversationnya mata kuliah speaking itu mau tidak mau harus langsung yah, karena ngga seru chattingan kayak WA.. kayak kita WA kan kurang efektif ya itu masalah sinyalnya. Banyak mahasiswa yang speakernya rusak, yang audionya tidak bisa seperti itu. Itu masalah pertama masalah sinyal itu maslaah klise yah, terus yang kedua saya itu tidak bisa maksimal menyampaikan materi yang perlu saya sampaikan pada sebuah mata kuliah seperti tadi misalnyakanji saya terpaksa tidak bisa mengajarkan cara menulisnya, terus untuk yang listening saya tidak bisa secara langsung mendengarkan audio kepada mahasiswa seperti yang saya lakukan pada saat kuliah tidak online, seperti itu.

Ya kendala-kendalanya seperti itu karena emang terbatas ya kita terbatas oleh jarak dan waktu file doang dan waktu haha.. oleh ruang bukan waktu.. kalau waktunya kan sama, oleh ruang kan terbatas oleh ruang kita hanya mengandalkan satu layar laptop kecil gitu kan jadi tidak bisa leluasa seperti pada saat kuliah online. Terus dari segi kesehatan juga kendalanya sekarang itu mata saya sekarang makin burem. Kita sehari habisin mata kuliah itu, tiap hari kan harus di depan layar kan ya jadi mau tidak mau kena radiasi padahal kacamata saya sudah radiasi loh.. udah anti radiasi. Itu tuh dikeluhkannya oleh semua dosen, dosen jepang juga ‘aduh mataku’ katanya ‘mataku sekarang udah mulai burem nih tiap hari manteng terus di depan layar’ seperti itu. Terus duduk terus, yang udah sepuh udah 40 tahun ke atas itu pinggangnya katanya duduk terus, seperti itu”

Peneliti : “Termasuk saya pak, saya sudah 40 lebih saya ngerasa capek kaki, leher .”.

Informan : “Oh iya pak? Suara juga, kan kita seperti penyiar radio ya kita hehehe..”

Peneliti : “terus secara psikis lagi pak..”

Informan : “secara psikis juga lho, apalagi yang pertama pertama itu termasuk yang lain juga mengeluhkan, oh tapi anehnya makin kesini kok makin terbiasa.. makin udah mulai enjoy.. use to be ya.. makin terbiasa dengan sistemnya MS teams, katanya sudah mulai enjoy.”

Peneliti : “Kalau bapak tadi, terkendala secara psikis tadi mulai terkurangi atau bagaimana pak?”

Informan : “oh iyaa.. itu Cuma awal-awal saja sih karena awal-awal kita terlalu semangat ya dengan MS Temas tapi lama kelamaan mungkin karena sudah terbiasa jadi untuk kalau misalnya saya kalau mata udah mulai lelah saya tidak akan memaksakan. Jadi saya cukup ee.. apa namanya ee.. dengan mengandalkan audio saja seperti itu, mata saya engga diarahkan ke layar seperti itu. Terus kalau saya sakit pinggang, saya sambil bersandar ngga.. ngga bakal keliatan sama mahasiswa kan itunya kan dimatikan hahaha visualnya dimatikan jadi ee.. seperti itu. Kesehatan kita kan nomor satu, jadi jangan sampai kesehatan kita menjadi terganggu gara-gara MS Teams ini, seperti itu.”

Peneliti : “Mohon maaf kalau saya tanya yang satu ini pak, kalau untuk kehidupan pribadi merasa terdampak ngga pak dengan kuliah online ini?”

Informan : “ee.. karena saya masih sendiri saja nyantai-nyantai saja sih, tidak. Tapi ini juga malah jadi suatu yang baru buat saya oh ternyata kuliah online itu menarik juga, ada sisi menariknya meskipun ada sisi lelahnya seperti itu. Kalau saya si anggap ee.. saya menganggapnya yang baru itu saya anggap sebagai suatu hal yang emang jadi pengetahuan baru bagi saya jadi.. pengetahuan baru yang emang sangat menarik sekali di jaman sekarang ini. Emang yang namanya kuliah online emang sudah menjadi suatu keharusan, bukan keharusan.. tapi sudah menajdi suatu hal yang wajar dilakukan kan memang di jaman modern seperti ini, jadi saya tidak ada beban sebenarnya. Jadi engga berpengaruh terhadap kehidupan pribadi saya, saya enjoy-enjoy saja hehehe..”

Penelit : “jadi misalnya kalau sama pak Rektor besok itu bahwa kuliah itu harus blended dalam arti harus mix berarti tenang-tenang saja ya pak ya..”

Informan : “hahaha secara sepihak si, soalnya saya tanya ke mahasiswa itu mereka kebanyakan mengeluh, dalam artian pengin segera kuliah normal. Karena menurut saya mereka merasa itu ee.. kuliah online itu meskipun ada satu dua mahasiswa yang suka, udah saya survey kebanyakan mereka ee..katanya tidak efektif, lelah, capek, terus kuotanya juga nyedot dan segala macamnya. Mereka kepengin kuliah seperti biasa di kelas, seperti itu.”

Peneliti : “Kalau bapak pribadi, akses ngga masalah ya?”

Informan : “Lancar banget akses segala macam lancar..”

Peneliti : “Mungkin ini yang terakhir pak, apa yang kira-kira menurut bapak dari pengalaman yang sudah bapak ceritakan itu yang perlu ditingkatkan pak? Mungkin ada satu dua hal yang kurang, tapi ‘saya bisa meningkatkan’ kira-kira apa ya pak?”

Informan : “Peningkatan dari segi metode pengajaran? dari segi fitur? atau dari segi..”

Peneliti : “bebas bisa menyampaikan dari sisi.. apakah dari sisi teknisnya MS Teams-nya atau dari sisi pengajarannya..”

Informan : “Saya pengin si MS Teams itu ee.. udah bagus ya.. tapi ada fitur-fitur lain yang lebih mempermudah pembelajar seperti saya misalnya, di MS Teams itu sya tidak tau apakah hanya PPT saja yang bisa ditampilkan ya.. selain PPT missal untuk yang lainnya ee.. kayak Word, Excel itu bisa engga ya. Terus yang kedua, audio.. biar kita pas ngajar listening bisa sambil mendengarkan audio secara langsung seperti itu. Terus ada juga mungkin.. MS Teams-nya bisa langsung konek ke suatu web, dibuka kan praktis ya.. engga harus di close dulu segala macamnya seperti itu.. mungkin agak sulit ya tapi itu harapan saya untuk mempermudah pengajar mengajar ini.. mengunakan MS Teams.

Terus peningkatan yang lainnya, ya dari segi metode pengajaran, seorang dosen.. pengajar.. harus lebih kreatif lagi memanfaatkan ee.. MS Teams ini dengan semaksimal mungkin sehingga materi yang kita sampaikan bisa tersampaikan dengan baik seperti kita mengajar pada saat di kelas, seperti itu. Tapi itu agak sedikit sulit ya.. karena emang keterbatasan ini media, seperti itu. Terus satu lagi, hasilnya juga sebenarnya ee.. apakah mempunyai dampak positif.. pas UTS kemarin dari segi nilainya itu emm.. apa namanya.. signifikan sekali peningkatannya itu karena mungkin pas UTS mereka tidak ada yang mengawas apa segala macam, jadi kelas yang bawah pun jadi A semua loh.. itu A semua loh. He eh.. itu kurang tau posistif atau negative, saya sebagai pengajar merasa senang ‘oh biasanya kalau kuliah di kelas mereka dapat nilai B atau C. Sekarang setelah online mereka dapat nilai A, Alhamdulillah saya ikut senang hehehe. Sebeum UTS pun kita bilang, jangan liat buku yaa… tapi ternyata engga tau mereka jujur tapi.. bulan puasa mereka akan jujur ngga nih .”

Peneliti : “Kalau keaktifan kelas selama di perkuliahan bagaimana melihatnya?

Informan : “keaktifannya ee.. kalau saya selalu membuat mereka harus aktif karena ada penambahan nilai yang saya masukkan, kadang diiming-iming nilai ya langsung mereka aktif sekali. Tapi kalau kita tidak iming-iming penambahan nilai segala macam, yang aktif hanya itu-itu saja seperti yang di dalam kelas. Tapi kalau kita.. saya biasanya sih ngasih semacam stimulus agar mereka bisa aktif seperti itu. Jadi kalau misalnya yang aktif yang bertanya saya kasih point 2 untuk tambahan UTS, terus mereka wah semuanya nanya jadi seperti itu untuk stimulusnya seperti itu..”

Peneliti : “Oh iyaiya.. Pak, untuk segi peningkatan tadi disebutin, ada ngga juga punya harapan nggak dari mahasiswa? Mahasiswa harus bagaimana..”

Informan : “Harapannya.. pas saya tanya tuh harapannya pengin segera berakhir katanya hahaha, anehnya ada beberapa yang suka juga ee.. ‘sensei kalau ini tidka banyak memakan kuota saya lebih suka online’ katanya ‘kita lebih kreatif juga mencari-cari em.. bahan-bahan sendiri seperti yang suruh sensei, jadi kita bisa leluasa eksplor pengetahuan luar yang bisa saya ajarkan’ karena kalau dikelas katanya kita disuapin satu persatu diajarkan grammar ini penggunaannya seperti apa. Jadi kalau misalnya engga online, mereka merasa lebih ini.. eh kalau online ya, mereka merasa lebih leluasa eksplor pengetahuan, seperti itu.”

Peneliti : “Kalau harapan ke mahasiswa, apa yang seharusnya mereka tingkatkan itu pak misalnya?”

Informan : “He em. Ee.. dengan online ini saya bisa sampaikan ke mereka ambil sisi positifnya jangan hanya ambil sisi negatifnya, sisi positifnya dengan online ini Anda bisa lebih mandiri, menjadi seoarang mahasiswa sebagai Student Center Learning tidak perlu disuapi satu-persatu oleh dosen. Sehingga ini untuk sofskill akan menumbuhkan softskill kemandirian itu yang pertama, yang kedua masalah kejujuran misalnya pada saat UTS, UAS, atau mengerjakan tugas silakan dilaksanakan sendiri, seperti itu. Jadi ini juga mengajarkan softskill kejujuran, kejujuran.. kemandirian, nah keterbukaan juga seperti itu dan usaha kreatif dan inovatif seperti itu. Jadi harapan saya ke mereka seperti itu, silakan Anda menjadi mahasiswa yang lebih mandiri, lebih jujur, lebih kreatif, ee.. karena emang seperti itu yang diharapkan mahasiswa itu. Tidak hanya sekadar disuapin satu persatu oleh senseinya seperti itu.”

Peneliti : “Menarik.. Saya bahkan belajar banyak ini dari bapak, sebelum saya akhiri apakah ada sesuatu yang lain yang mungkin pengin disampaikan?”

Informan : “Boleh.. ee.. untuk semester depan Wakil Rektor mengharapkan bahwa meskipun misalnya ee.. pandemi sudah berakhir dan kita kuliah seperti biasa tapi ada beberapa mata kuliah maksimal berapa persen yah.. kita harus menggunakan kulon bukan MS Teams. Nah harapan saya, ee.. Wakil Rektor.. rektorat.. tidak hanya sekadar untuk mengasih intruksi tapi juga harus ee.. istilahnya apa ya.. ee.. istilahnya harus mempersiapkan segalanya dengan baik dan benar. Seperti misalnya, kulon itu kan lebih ribet ya.. lebih ini dari pada MS Teams ya seperti itu.. terus dosen-dosen di tempat saya juga sudah mulai merasa ini.. di Jepang juga.. ada gak sih kulon, kan kulon katanya engga ada interaksi segala macamnya, kalau ngajar speaking, kalau ngajar listening apa bisa bagi kulon.

Tapi itu satu hal yang bagus akan kita support, jadi untuk mata kuliah –mata kuliah yang nanti yang bisa menggunakan kulon akan kita laksanakan, tapi mungkin perbaikan fitur-fitur di kulon yah biar lebih efektif ee.. biar lebih nanti saat digunakan itu bener-bener manfaatnya ada gitu kan. Jadi hasilnya sangat efektif dan tidak merugikan mahasiswa dan kita juga sebagai dosen, seperti itu. Jadi kita support apa.. harapan saya si seperti itu ke rektorat, itu kulon bisa boleh tapi fitur-fiturnya dan segala macamnya untuk lebih diperbaiki, dimodernisasi seperti itu, itu harapan ke rektorat ya..”

Peneliti : “Siap pak semoga pihak teknisi di rektorat juga mempersiapkan dengan lebih pas..”

Informan : “iya itu.. teknisinya.. he em teknisinya..”
