# Supplementary material for: Lecturers’ information literacy experience in remote teaching during the COVID-19 pandemic
Source: PLoS One. 2022 Mar 18;17(3):e0259954. doi: 10.1371/journal.pone.0259954 (PMC8932599; doi:10.1371/journal.pone.0259954)
Supplement: S4 File — (DOCX) [file pone.0259954.s004.docx]

**TRANSKRIP INFORMAN KETIGA**

Peneliti: Pak semester ini ngajar berapa SKS, Pak?

Informan : Saya mungkin kalau SKS ada sekitar 14 ya karena ada 7 kelas masing-masing 2 SKS. Jadi ya 14 SKS.

Peneliti : Mengajarnya menggunakan teams atau ada menggunakan aplikasi lain pak?

Informan : Untuk aplikasi utama saya menggunakan teams, sesuai yang direkomendasikan universitas. Tetapi beberapa kali sempat misalkan meng-*upload* file lewat kulon itu tadi hanya saja sampai sekarang memang secara aktif belum saya gunakan karena satu dan lain hal termasuk belum begitu familiar dengan sistem yang ada di kulon. Hanya lebih kepada saya meng-*upload* file sebagai instruksi tindak lanjut dari instruksi rekstor kemarin, kemudian aplikasi sehari-harinya menggunakan teams itu pak.

Peneliti : Ada kesulitan nggak pak dalam memakai teams?

Informan : Sejauh ini saya kira secara teknis kesulitan yang sering saya alami adalah ketika *loading* aplikasi itu sendiri, mungkin lebih berkaitan dengan kapasitas di laptop saya itu pak. Jadi kalau untuk fitur dan program-program saya kira tidak ada masalah tapi hanya beberapa kali karena mungkin pda saat yang sama saya membuka Google, video mungkin, teams itu jadi *crash* atau harus *loading* lama itu mungkin, itu aja kesulitannya pak.

Peneliti : Itu masih dialami pak?

Informan : Masih dialami tapi dibeberapa waktu sjaa, kadang kala lancar kadang kala tidak. Lebih kepada saat saya membuka pertama kali teams itu, mungkin *loading*-nya kan butuh memori yang lebih, itu kendalanya pak.

Peneliti : Selama ini mengajarnya saat berinteraksi dengan mahasiswa seringnya bagaimana pak caranya?

Informan : Saya terus terang, selama ini membagi menjadi beberapa metode ketika aplikasi di kelas itu. Jadi, pertama saya *brainstorming* menggunaan chat, jadi saya akan meminta mahasiswa untuk membaca sebuah artikel yang saya instruksikan secara tertulis melalui chat. Tetapi setelah itu, saya akan mealokasikan kira-kira kurang lebih 1 jam atau mungkin 40 menitn ya atau 40-60 menit untuk sesi *video live* interaksi seperti itu. Jadi, artinya ketika saya meminta mahasiswa untuk membaca saya tidak akan membuat mereka menghabiskan banyak kuota hanya untuk membaca aja, tetapi saya maskimalkan secara interaktif di sesi yang kedua.

Peneliti : Selama ini lancar pak berinteraksi dengan mahasiswa?

Informan : biasanya *trouble*-nya klasik pak, jaringan yang *up and down* gitu pak. Jadi baik dari saya maupun dari mereka itu yang paling sering si, tetapi dalam *upload* materi, pemanfaatan fitur saya kira secara dasar sudah saya kuasai.

Peneliti : Saat meng-*upload* materi atau saat menyampaikan materi itu apakah itu materi yang sudah pernah digunakan sebelumnya sebelum pandemi ini, atau bagaimana pak?

Informan : Saya justru belajar dari kemarin pak. Jadi semester sebelum pandemi itu saya merasa organisasi materi itu lebih tidak terstruktur sehingga saya berpikir karena pandemi ini harus menggunakan fitur online, saya membuat sesuatu yang harusnya saya lebih terstruktur karena untuk memudahkan mahasiswa. Jadi bisa dikatakan materi yang saya gunakan adalah materi-materi baru begitu yang belum pernah saya ajarkan sebagian besarnya. Karena saya berusaha untuk menyusun *lesson plan*, menyusun materi itu sebaik mungkin untuk mengindari misalnya mahasiswa yang kesulitan jaringan itu tadi untuk bisa memanfaatkan sendiri dari arahan-arahan yang saya *upload* itu pak.

Peneliti : Apakah materi-materi itu tersedia di teams pak buat mahasiswa?

Informan : Ya, saya selalu membuat sebuah folder khusus setiap minggunya itu saya beri judul *week one*, *week two* atau *class one* atau *class two* untuk membuat mahasiswa mengakses itu secara terbuka. Jadi baik file berupa pdf, word, power point atau kemudian tautan tertentu saya masukkan kesitu.

Peneliti : Terkait materi baru pak, lancar nggak selama ini pak untuk mempelajari bagaimana cara meng-*upload* lalu bagaimana cara *sharing* dengan mahasiswa pernah ada kesulitan lancar-lancar saja atau bagaimana pak?

Infoman : Bisa dikatakan 80% lancar kecuali faktor teknis yang berhubungan dengan jaringan, koneksi, laptop yang lambat.

Penelitian : Mencari selama materi baru itu bagaimana pak? Bisa diceritakan misalnya kan ini jamannya online semua ya materi mungkin dari berbagai format, dan untuk disajikan ke mahasiswa itu mencarinya bagaimana pak?

Informan : Yang pertama mungkin gini ya pak, karena ini kebetulan di bidang saya adalah *language skills* dan juga ada juga yang materi sifatnya teoritis seperti pengkajian budaya saya bagi menjadi dua jenis itu dulu ya tipe. Pertama yang kalau materi sifatnya *language skills* itu saya memanfaatkan beberapa fitur penyedia konte-konten digital seperti katakanlah yang paling sering saya gunakan adalah Youtube untuk kemudian mengakses audio file saya unduh begitu kemudan saya *convert* kedalam mp3 saya *upload* ke grup ke teams itu sendiri.

Yang kedua saya memang sudah punya bahan ajar yang sejak lama tersimpan di laptop saya itu file-file digital seperti [nama materi] ataupun beberapa materi terkait lain. Selain itu saya juga mengakses beberapa artikel dari sebuah laman tertentu katakanlah misalkan *bbclearning.com* gitu untuk materi yang berupa artikel berupa bacaan, atau kemudian kadang kala juga ada *Cambridge* itu ataupun *web addres* yang memang memuat konten-konten pembelajaran bahasa inggris, itu yang *language skills*.

Kemudian yang sifatnya itu teori beberapa karena saya berpegangan pada RPS dan selalu berusaha mencari referensi yang tertulis di RPS pak, misalkan katakanlah materi kajian budaya atau komunikasi lintas budaya jadi merupakan mata kuliah pertama yang ada di sastra inggris, pertama kali dalam arti diajarkan karena itu kurikulum baru begitu dan beberapa materi beruntungnya itu mudah didapatkan melelui beberapa penyedia e-book, digital book seperti e-pdf yang sepertinya illegal ya tapi juga ada saya jumpa yang seperti *epdf.pub.app*, e-pdf drive atau kemudian secara langsung saya menggunakan *open access* yang disediakan oleh universitas katakanlah yang pernah saya gunakan untuk men-*download* adalah JSTOR itu biasanya digital seperti itu pak.

Peneliti : Pak tahunya itu dari mana pak? Khususnya yang bukan dari [nama universitas]

Informan : Saya sebenarnya mendapatkan tradisi tradisi semacam itu sudah saya dapat ketika saya S2, jadi dari antar teman kemudian informasi yang disediakan dosen pada saat itu sehingga dasarnya sudah dapat

dan setelah itu saya lebih bermain ke kombinasi *keyword* mungkin. Jadi misalkan situs yang paling illegal yang saya tau adalah *bookfield* itu yang paling populer ketika saya S2, nah saya kan taunya itu ada *bookfield* kemudian ada heading ini adalah *free e-book*, kemjdian saya coba kombinasikan itu di mesin perncari *e-book* gratis atau *free e-book* download, dan nemu banyak.

Peneliti : Pernah menyajikan *e-book* ke anak-anak atau hanya link ke *e-book* itu pak?

Informan : Ketika saya mempresentasikan materi itu lebih kebanyakan menggunakan *e-book*-nya langsung dari pada saya mengarahkan mahasiswa untuk ke link tersebut begitu, karena menjaga *image* ya mungkin.

Peneliti : Pak sebagai dosen, merasa penting nggak pak buat Pak bahwa mahasiswanya Pak juga memiliki ketrampilan yang sama dalam mencari informasi pak?

Informan : Ya, sangat penting menurut saya. Jadi, tetapi saya kira sampai saat ini yang saya lakukan adaah dengan agak berhati-hati pak ketika memberikan informasi mengenai bagaimana mengakses *study resources* karena saya khawatir kemudian ini akan menjadi beretensi buruk, dari dosen yang pernah saya baca beberapa komentar mahasiswa terkait perilaku pencarian informasi dosen yang mungkin bisa dikatakan saya pernah berbicara *statement* seorang literer begitu ya misalkan katanya [nama universitas] gitu tetapi kok nyari informasinya kok di sini, nah saya ingin menghindari ini juga semacam itu. Jadi saya langsung arahkan ke langsung ‘ini bukunya seperti ini’

tapi dalam matakuliah seminar yang notabene mahasiswa sudah tingkat lanjut begitu saya berikan beberapa edukasi materi ya secara khusus bagaimana perilaku pencarian informasi itu tadi, misalkan dengan memanfaatkan *open access* yang diberikan oleh [nama universitas] melalui repository melalui jurna-jurnal yang dilanggan [nama universitas] atau beberapa situs yang memang yaa mungkin bisa dikatakan cukup lengkap lah *e-book* itu sendiri.

Peneliti : Disela-sela perkuliahan, apakah juga Pak memberikan *tips and trick* untuk mencari informasi itu atau Pak percaya aja mereka bisa sendiri mencari?

Infroman : Saya lebih tergantung mata kuliah pak, biasanya mata kuliah yang intensif untuk kemudian ada sesi seperti itu yaitu pencarian informasi atau bagaiaman, ke mata kuliah yang berhubungan dengan *language skills* terutama beberapa materi seperti *interpeting* atau penerjemahan ataupun beberapa materi terkait fonologi yang biasa saya.. karena apa, karena kalau kaitannya dengan *interpreting* atau *translation* itu kita akan membutuhkan sesi di mana kita harus mengecek betul apakah sebuah kata itu lazim atau tidak digunakan oleh orang diluar atau kita sebut sebagai leak dalam korpus nah itu saya betul-betul ajarkan ke mereka bagaimana langkah-langkahnya misalkan saya harus mencari tahu apakah kata *velocitize* itu bisa *equal* dengan penerjemahan sodara sebangsa gitu. Nah saya akan mengecek mengarahkan mereka ke sebuah mesin korpus atau kamus atau Google secara umum ‘oh ini nggak layak’. Jadi tergantung mata kuliahnya pak.

Peneliti : Cara itu Pak terapkan baru selama pandemi atau sebelumnya sudah?

Informan : Sebelumnya sudah, karena kebetulan saya *interpreting* itu selalu dapet kelas jadi mungkin kayaknya tiga tahun belakangan saya menggunakan itu dan saya justru suka sesi itu karen itu *killing time* betul dari pada saya harus nyrocos yang teoritikal itu saya ingin praktik langsung ini itu *killing time* karena .. waktu untuk ngetik karena mahasiswa bisa bertanya interaktif gitu pak.

Peneliti : Itu digunakan juga pak saat sebelum pandemic ini?

Informan : Iya, digunakan saat mata kuliah *interpreting* terutama

Peneliti : Bagaimana cara mereka inget? ..

Informan : Mereka menggunakan handphone, smartphone mereka sendiri.

Peneliti : Dan itu lebih efektif selama pandemic ya?

Informan : Saya kira begitu pak,

Peneliti : Yang paling membedakan apa si pak? Perbedaan mengajar saat tradisional di kelas sama sekarang saat online seperti ini pak?

Informasi : Itu bisa lebih, menurut saya elaboratif dalam mencari materi dan dalam memanfaatkan metode-metode lain gitu dari pada sekadar ceramah saja, ada sesi-sesi di mana kita itu punya kebebasan sendiri tidak merasa diawasi dosen terus atau dosen diawasi mahasiswa terus untuk ada ruang untuk membaca konten tertentu atau lain lain disarankan itu.

Peneliti : Apakah maksud elaboratif itu berarti lebih banyak variasi atau bagaimana pak?

Informan : Lebih banyak variasi pak.

Peneliti : Pernah ada komen yang khusus menurut Pak dari mahasiswa selama ini terkait dengan pembelajaran online ini? Misalnya apakah lebih mudah memahami materi ketika online atau mungkin sebalikannya atau bicaranya teralu cepet ketika di video atau apapun itu pak?

Informan : Saya pernah menanyakan beberapa kali tapi nampaknya mahasiswa belum terbuka ya karena itu kebetulan sesi itu saya tanyakan *live-call* atau video seingga lebih banyak yang saya dengar adalah ucapan terimakasih di akhir episode mengajar itu tadi. Tetapi pertanyaan itu sering saya lontarkan dengan pertanyaan *‘how do you enjoy the online classes?’* ataupun ‘bagaimana sih anda dealing dengan situasi semacam ini’ itu pernah saya tanyakan.

Peneliti : Sejauh mana kepuasan Pak dalam mencari materi misalnya juga termasuk dalam menggunakan berbagai sumber informasi yang ada untuk mencari informasi buat mahasiswa itu, Pak sudah sejauh mana apakah puas seperti apa puasnya atau masih belum maksimal atau mungkin bagaimana?

Informan : Saya rasa masih belum maksimal dalam arti selama ini kita terbiasa dengan pola pengajaran *offline* begitu bertatap muka langsung di mana kita sering dihadapkan dengan sumber-sumber tertulis misalnya dengan mudah kita bisa akses di perpustaaan prodi dan itu sesuai dengan RPS yang ada. Sementara ketika kita harus mengadaptasinya kedalam online itu tidak selalu apa yang tertulis apa yang ada di dokumentasi di sumber tertulis itu bisa dengan mudah kita dapatkan secara online, karena minimnya workshop yang kemudian digelar secara formal oleh prodi ataupun oleh institusi yang berwenang. Sehingga kadang ada pertanyaan juga apakah sumber yang saya gunakan ini *reliable* atau tidak dari sisi perkembangan ilmu pengetahuan itu sendiri.

Peneliti : Ada upaya khusus pak untuk mengatasi itu mungkin?

Informan : Saya lebih sering menggunakan Youtube itu sendiri untuk menilai apakah materi yang saya gunakan itu kemudian sesuai dengan beberapa kurikulum yang digunakan oleh satu institusi pendidikan bahasa inggris tertentu. Jadi saya misalkan saya me-*refer* pada *bbclearning.com* atau *britisth council* karena mereka sudah punya rekam jejak yang lama, ketika saya menyampaikan materi A saya googling atau saya lihat di Youtube atau situs mereka kok pembahasannya sejalan atau Cambridge atau oh berarti ya mungkin paling nggak ya mendekati saya lihat seperti itu kayak saya biasanya.

Peneliti : Pak memilih itu karena sejak mengajar online ini atau itu karena ya udah normal saja sejak dulu penggunaan youtub penggunaan bbc?

Informan : Saya kalau bbc dan voa atau Cambridge itu udah sejak lama, jadi memang itu sudah saya jadikan referensi utama untuk pengajaran bahasa itu sendiri. Tetapi kekhawatiran bahwa kemudian itu valid atau tidak itu lebih tinggi ketika ini, karena saya lihat kan semua serba terbuka ya *open access* itu jadi ada kekhawatiran juga kemudian komentar-komentar yang negatif itu kemudian menyasar ke materi-materi itu. Karena kan lebih mudah diakses oleh banyak orang.

Peneliti : Pak pernah menyampaikan itu ke anak-anak nggak pak, kekhawatiran itu pak?

Informan : Belum pernah, saya by pass. …

Peneliti : Menurut Pak itu, seberapa penting mahasiswa itu membaca jurnal pak, maksud saya mungkin tadi Pak menyebut mata kuliah seminar ya apakah Pak merasa mahasiswa itu penting membaca jurnal atau cukup materi-materi yang dari Pak sendiri?

Informan : Menurut saya wajib pak, membaca jurnal hukumnya bagi mahasiswa atau dengan kata lain sangat penting karena saya sendiri sering membuat *disclaimer* ke mahasiswa bahwa apa yang saya sampaikan ini bisa jadi adalah setitik dari sekian luas lautan keilmuan yang ada, sehingga Anda bisa jadi lebih tahu dari saya dan saya menerima itu, saya sering kali melakukan seperti itu. Apalagi kalau kaitannya dengan mata kuliah seminar ini kan mahasiswa lebih elaboratif ya lebih variatif dalam penyajian topik gitu dan saya bahkan kadang kala asing sekali dengan apa yang disampaikan oleh mahasiswa. Nah untuk mendapatkan informasi lebih atau teori yang berkaitan dengan mahasiswa, tentu membutuhkan sumber-sumber yang baik itu *updated* atau *reliable* secara keilmuan itu dengan mngacu pada sumber-sumber yang terpercaya yang dikelola oleh misalkan univeristas melalui *open access* jurnal itu tadi.
