# Supplementary material for: Lecturers’ information literacy experience in remote teaching during the COVID-19 pandemic
Source: PLoS One. 2022 Mar 18;17(3):e0259954. doi: 10.1371/journal.pone.0259954 (PMC8932599; doi:10.1371/journal.pone.0259954)
Supplement: Attachment — Submitted filename: Response to reviewers.docx [file pone.0259954.s005.docx]

**Response to REVIEWERS**

| Authors | Heriyanto; Lydia Christiani; Rukiyah |
| --- | --- |
| Manuscript ID | PONE-D-20-39011 |

| Editor Remarks | Authors Comments |
| --- | --- |
| 1. Please ensure that your manuscript meets PLOS ONE's style requirements, including those for file naming 2. Please provide additional details regarding participant consent. In the ethics statement in the Methods and online submission information, please ensure that you have specified (1) whether consent was informed and (2) what type you obtained (for instance, written or verbal, and if verbal, how it was documented and witnessed). If your study included minors, state whether you obtained consent from parents or guardians. If the need for consent was waived by the ethics committee, please include this information. 3. When reporting the results of qualitative research, we suggest consulting the COREQ guidelines: <http://intqhc.oxfordjournals.org/content/19/6/349>. In this case, please consider including more information on the number of interviewers, their training and characteristics. Moreover, please describe in more detail the methodology used for data analysis, and please provide the interview guides used as a Supplementary file. | The manuscript has been revise following PLOS ONE’s style requirement as stated in <https://journals.plos.org/plosone/s/file?id=wjVg/PLOSOne_formatting_sample_main_body.pdf> and  <https://journals.plos.org/plosone/s/file?id=ba62/PLOSOne_formatting_sample_title_authors_affiliations.pdf>  Information about participant consent has been provided in the ethics statement in the Methods and online submission form. The written consent was informed to the participants when the participants agreed to be interviewed and before they were interviewed.   - Additional information about number of interviewer, the interviewer training and characteristic have been provided following COREQ guidelines - The data analysis method has been described in details in the Methods section. Thematic analysis was employed in the study to interpret the qualitative data obtained from the interviews. - The interview guide is provided as a supplementary file. |
| Reviewer 1 Remarks | Authors Comments |
| - I would have liked to know more about how these lecturers were recruited; also it would have been good to have more information on each lecturer - such as years teaching, age, comfort with technology. - I also suggest a more thorough literature review. Other research exists in the area of teaching online, though perhaps without the focus on information literacy. - I also question your use of thematic analysis. From my own experience, thematic analysis does not include the use of codes. I am also not seeing any explanation of how you derived those specific codes. - Your findings are very interesting but some parts are vague. For example, "some lecturers commented that 30-minute lectures were at times to short," "most lecturers encouraged students to allocate their own timeframes," "the lecturers found that they could encourage self-study..." Can you be more specific with these findings? What do you mean by "most," or "some?" Did all lecturers find that they could encourage self-study? - Last, there are grammatical errors throughout, so this needs to be edited. | The participants recruitments technique and additional information about each lecturer have been added to the Methods section  Thank you, this is really great suggestion. We have followed this suggestion by providing research in the area of emergency remote teaching. These researches considered relevant to this study as the context of the study is emergency remote teaching and information literacy.  This study employing thematic analysis introduced by Braun & Clarke which it involves generating codes as its second phase for the data analysis. The details of Braun & Clarke publication on thematic analysis that employed in this study is available here Virginia Braun & Victoria Clarke (2006) Using thematic analysis in psychology, Qualitative Research in Psychology, 3:2, 77-101  The sentences have been revised with the suggested wording to be more specific with the participants.  The revised version of the manuscript has been proofread by professional proofreading services to improve the clarity and readability. |
| Reviewer 2 Remarks | Authors Comments |
| - I'm not sure you've sufficiently demonstrated why this is connected to information literacy. Although I appreciate the literature review, this doesn't highlight any formal standards or frameworks (e.g. IFLA or, in the U.S. context ACRL). It seems to me what you're describing is information behavior of lecturers? That would require a different lit review - I wasn't entirely clear on the research questions - I know you were investigating how the lecturers changed their teaching based on the transition to online education, but it wasn't clear to me how this fits into the overall research framework of either information literacy or online teaching. Again, for the latter, a different or supplemental lit review and framework would be needed - How do these particular lecturers represent the wider population? What were your questions? Methods need to be more specific. | This study employed the relational perspective of information literacy which emphasis the experience of individuals with their information world. This experience would enable them "to draw upon in new situations", which can be translated as the way they learnt from that situation and applying them to a new situation. Bruce described the information literate person as being “one who experiences information literacy in a range of ways, and is able to determine the nature of experience it is necessary to draw upon in new situations” (1997a, p. 169). Therefore, information literacy is connected to this new situation experienced by the lecturers of this study. Moreover, the relational perspective of information literacy framework is adopted in this study and have been mentioned in the literature review section.  The literature review section has been revised as suggested emphasizing the emergency remote teaching and information literacy framework.  This qualitative study revealed the perspective of lecturers in the Faculty of Humanities, Universitas Diponegoro regarding their online teaching experience. The findings of the study do not aim to generalize the whole population of lecturers. However, the Methods section has been revised as suggested by providing research question |
