# Supplementary material for: Lecturers’ information literacy experience in remote teaching during the COVID-19 pandemic
Source: PLoS One. 2022 Mar 18;17(3):e0259954. doi: 10.1371/journal.pone.0259954 (PMC8932599; doi:10.1371/journal.pone.0259954)
Supplement: Attachment — Submitted filename: Response to reviewers 2.docx [file pone.0259954.s006.docx]

**Response to REVIEWERS**

| Authors | Heriyanto; Lydia Christiani; Rukiyah |
| --- | --- |
| Manuscript ID | PONE-D-20-39011 |

| Reviewer 1 Remarks | Authors Comments |
| --- | --- |
| I appreciate the changes to this manuscript. My concerns were addressed and the research question more clearly answered. While the data support the conclusion, there were instances where only one participant's concerns were addressed. It would have been helpful to see the questions from semi-structured interviews to get a better perspective. Overall, however, I believe this manuscript is suitable for publication. Thank you for your extensive revisions! | Thank you for the suggestion. We have followed this suggestion by providing the questions from semi-structured interviews in the method section. |
| Reviewer 3 Remarks | Authors Comments |
| Firstly, as reviewer 2 remarked, a much stronger conceptualization of information literacy must not only be developing in the introductory pages of the manuscript, but it must also be shown to have clearly guided the data analytic procedure. A good conceptual definition, at the very least, should provide a guideline for what DOES count as information literacy, as well as a clear demarcation of what is NOT information literacy. The relational framing provided, while interesting, is too broad to provide inclusion and exclusion criteria that will help focus and develop rigor in the data analysis  On a related note, the discussion of "qualitative research" in the methodology is also quite broad; in some cases, this broadness is a mischaracterization of the diversity of methodological thought within research traditions who have been historically grouped together as "qualitative" (e.g. discourse analysis, grounded theory, phenomenology, narrative analysis, etc.) but are, in reality, founded in very different epistemological and ontological assumptions. My advice would be to clarify the particulars of the qualitative approach that you are taking (the thematic approach you refer to seems to draw on certain traditions of grounded theory, or at the least, constant-comparative analysis), but you should make these distinctions more clear in your discussion  Finally, related to the comments of other reviewers, one challenge in presenting thematically analyzed research is making transparent the rigor and systematic nature of your analysis. If this is not clear to readers (or reviewers), then the findings of your work are called into question. My suggestion here would be to provide more clear examples of how you developed codes from your open readings of the data (stage 1), how those codes were revised, refined, and reapplied to the data (stage 2), and how those codes where them "thematized" to provide your findings (stage 3). It would help, in this case, to make some of your own assumptions and prior experiences (what some refer to as positionality) as interpretive researchers transparent so as to increase readers' ability to evaluate your claims and their subjectivities. In these cases, drawing on passive constructions of language in the hope of maintaining "objectivity" actually serves to obscure, rather than clarify your analysis | Thank you for the suggestion. A more specific conceptual definition of relational perspective information literacy has been added to the literature review section as recommended.  Agree. This study employed basic qualitative approach following the recommendation by Merriam (2009). As the characteristic of basic qualitative is similar with all of qualitative research, other types of qualitative studies have additional dimension. For example, a grounded theory study seeks not just to understand, but also to build a substantive theory about the phenomenon being studied.  Thank you for the valuable suggestion. A detailed description of thematic analysis has been added to the method section for making transparent the rigor and systematic nature of the data analysis. |
