# Supplementary material for: Lecturers’ information literacy experience in remote teaching during the COVID-19 pandemic
Source: PLoS One. 2022 Mar 18;17(3):e0259954. doi: 10.1371/journal.pone.0259954 (PMC8932599; doi:10.1371/journal.pone.0259954)
Supplement: Attachment — Submitted filename: Response to reviewers 3.docx [file pone.0259954.s007.docx]

**Response to REVIEWERS**

| Authors | Heriyanto; Lydia Christiani; Rukiyah |
| --- | --- |
| Manuscript ID | PONE-D-20-39011 |

| Editor Remarks | Author Comments |
| --- | --- |
| Please review your reference list to ensure that it is complete and correct. If you have cited papers that have been retracted, please include the rationale for doing so in the manuscript text, or remove these references and replace them with relevant current references. Any changes to the reference list should be mentioned in the rebuttal letter that accompanies your revised manuscript. If you need to cite a retracted article, indicate the article’s retracted status in the References list and also include a citation and full reference for the retraction notice. | The reference list has been reviewed. As suggested, I have not cited the retracted paper. However, I have removed the preprint paper and replaced it with a relevant paper. The new paper listed in the reference list is   - Pal D, Vanijja V. Perceived usability evaluation of Microsoft Teams as an online learning platform during COVID-19 using system usability scale and technology acceptance model in India. Child Youth Serv Rev [Internet]. 2020;119(October):105535. Available from: https://doi.org/10.1016/j.childyouth.2020.105535 |
| Reviewer 1 Remarks | Author Comments |
| 1. The first sentence of the introduction needs a citation. 2. In the literature review, it says that the current study investigated the subject matter, rather than issues. Then later it says they are investigating the lecturers’ information literacy experience. This doesn’t make sense to me. 3. The authors frequently refer to the perspectives of the “majority of lecturers.” Why not give exact numbers since there were only 15 participants? 4. I’m confused by your statement that lecturers invited students to text discussions after video meetings. Do you mean text questions? Then you stated that students exhibited various behaviors during the online session. Can you be more specific? What behaviors do you mean? If you are referring to whether or not they participated in the chat or asked questions, you should specify that. 5. While you used thematic analysis for your methodology, I’m not seeing any clear explanation of themes. You describe the perspectives of individual or “majority” of lecturers, but do not connect those to any type of theme. I do not see the results to be significant since they seem to align with past research. 6. Last, there are many grammatical errors throughout, possibly because of translations. However, these errors make the manuscript difficult to follow. | 1. Thank you for raising this point up. The sentence has been revised and a relevant citation has been added. 2. Yes, I agree with the reviewer that the statements are a bit confusing. The first statement has been revised in order to cohere with the second statement that the study is about investigating lecturers’ information literacy experience. 3. The word ‘majority’ has been replaced with the exact name or number of the participants 4. I read the sentence again and I agree with you that this sentence should be more specific. More specific statements have been provided to the paragraph. It explains the behavior of students during the online conversation and text discussions, where some students contribute actively during the video meeting but some prefer to be active in the text discussion. 5. A description of each theme has been provided to the results section. Further, the significance of the results has also been provided to the discussion section. 6. The revised version of the manuscript has been proofread by professional proofreading services to improve the clarity and readability. |
